# Influence of Waste Basalt Powder Addition on the Microstructure and Mechanical Properties of Autoclave Brick

**DOI:** 10.3390/ma16020870

**Published:** 2023-01-16

**Authors:** Paulina Kostrzewa-Demczuk, Anna Stepien, Ryszard Dachowski, Rogério Barbosa da Silva

**Affiliations:** 1 Department of Building Engineering Technologies and Organization, Faculty of Civil Engineering and Architecture, Kielce University of Technology, al. Tysiąclecia PP 7, 25-314 Kielce, Poland; 2 St. Department Heitor Alencar Furtado, Federal Technological University of Paraná, 5000-Curitiba-Paraná, Curitiba 81280-340, Brazil

**Keywords:** basalt powder, sand-lime products, silicate bricks, autoclaving, post-production waste

## Abstract

In the production of building materials, there has been an increased interest in the use of by-products and industrial waste in recent years. Such modifications make it possible to solve not only technical and economic problems, but also environmental problems. This article describes the use of basalt powder waste in sand-lime products (silicates). The aim of the study was to manage basalt powder waste and to investigate the changes it causes in sand-lime products. The article describes the planning of the experiment, which directly determines the number of samples and their composition, which was necessary to conducting a full analysis and correctly illustrating the relationships occurring in the samples. Basic tests were carried out: compressive strength, density and water absorption, as well as optical tests and scanning microscopy. Based on the research conducted, it was concluded that the use of basalt powder as a component of sand-lime products has positive effects. Studies show that the best results are achieved with a proportion of powder in the raw material mass of about 10%—the compressive strength reaches almost 30 MPa, which is almost twice that of traditional silicate.

## 1. Introduction

Construction is a sector of the economy that causes a heavy burden on the environment, alongside other industries. Proper management of waste building materials is not only a serious logistical problem, but also a pressing social problem. This involves both restrictions on waste storage sites and stringent environmental standards related to the quantity and quality of waste generated. One of the ways of managing waste and by-products is their recycling and use for the production of building materials, which is consistent with the principle of sustainable development [1]. In the production of building materials, there has been an increased interest in the use of by-products and industrial waste in recent years. Such modifications make it possible to solve not only technical and economic problems, but also environmental problems. Mining waste [2] and industrial waste have been used in the modification of sand-lime products. The main purpose of these modifications is not only to reduce the cost of manufacturing products, but above all to care for the natural environment through waste management and energy saving at the production stage. Thanks to these modifications, products are created that are substitutes for previously used materials with similar physical and mechanical properties.

Autoclaved sand-lime bricks have been produced for decades. Traditionally, the ingredients of calcium silicate products are mainly sand, lime and water. The quality of the ingredients used, as well as the conditions of their production, translate into the final properties of autoclaved products. The theoretical composition of the raw material mixtures for autoclaved calcium silicate products is 92% quartz sand and 8% quicklime, which results in a molar ratio of C/S = 0.09 (C/S is CaO/SiO_2_ ratio). The proportions presented apply to mixtures that consist of quartz sand with 100% SiO_2_ content and lime with 100% reactivity. Research conducted in industrial silicate production plants shows that the conditions presented above almost never occur in reality. This is due to the use of sand of natural origin for the production of silicates (usually locally sourced, containing various impurities) and quicklime obtained in industrial plants, where it does not reach 100% reactivity. Accordingly, the actual composition of the autoclaved calcium silicate products is different from the theoretical composition mentioned above. When designing a mixture, it is important to select the mutual proportions of the components, taking into account their reactivity, so that the molar ratio of 0.09 is maintained.

Lime is the binding substance for calcium silicate products. Literature [3] indicate that with the increase in the content of lime in silicates, their compressive strength increases. From an economic point of view, lime is the most expensive component of the mix and in order to optimize the production and price of silicate bricks, the content of 10% of this component in the mix is not exceeded. The suitability of lime for the production of silicates is specified in the PN-EN 459-1 standard [4]. The PN-EN 459-1 standard defines unslaked ground quicklime with the symbol CL (90, 80, 70) as useful (with the content of CaO + MgO in the range of 70–90%, MgO below 5%, CO_2_ within 4–12% and SO_3_ below 2%). Lime should be slaked at a temperature of at least 60 ℃ for 10–30 min. Another important aspect related to lime is its granulation, expressed by the size of residues on sieves with a square mesh size of 0.09 mm (proportion not greater than 7%) and 0.2 mm (proportion not greater than 2%) [5].

Sand is the main component of autoclaved bricks, which is related to its relatively low cost and the chemical reactions that take place in the sand-lime mixture under appropriate conditions. The result of these reactions is the production of artificial rock, a very durable material that can be freely formed in the autoclaving process. Quartz sand affects the quality of the final autoclaved product. In addition to the SiO_2_ content, the shape, size and mineral content of the sand can also affect the quality of silicate bricks. The use of sands with a silica content of less than 80% is avoided. Quartz sands used for the production of silicate autoclaved products should not contain alkalis (Na_2_O and K_2_O) above 0.5%, Al_2_O_3_ content above 5%, Fe_2_O_3_ above 1.5% and MgO above 3% [5]. Silicate bricks are produced from semi-dry sands, which makes it necessary to ensure the appropriate granulation of the masses used for production. This ensures the appropriate degree of mass compaction and the subsequent strength of silicate products. Quartz sand should be characterized by a continuous grain size curve, without the dominance of any fraction. Proper graining of quartz sands used in the technology of sand-lime products is considered sand containing the following grain fractions: 2.5–0.5 mm in a proportion not exceeding 30%, and 0.5–0.05 mm in a proportion of at least 65%. Generally speaking, the sands used for the production of silicates belong to the group of fine-grained sands.

Natural sand is one of the main components of building wall materials (bricks, mortars and concrete mixes). In many countries, there is currently a significant shortage of natural sand of appropriate quality that can be used for the production of concrete [6]. In 2022, the prices of natural resources in Poland increased significantly, even by 30% [7]. This is mainly due to the crisis related to the COVID-19 pandemic and the war in Ukraine. The prices of energy, raw materials and fuels increased, and thus also the cost of transport and production. A similar situation is observed around the world. Therefore, other materials are being sought that may be a partial replacement for sand. The use of various types of industrial waste in the production of concrete not only reduces the consumption of natural resources, but also allows for the management of waste, which in many cases is burdensome for the environment. The literature is full of examples of the use of waste dust of marble [8,9], quartz [10], basalt [11,12,13,14], granite [15] or lime [9,10] as a substitute for sand in concrete. Waste basalt powder has been used in laboratory scale tests as a partial replacement of sand in the proportion of 0–30% of the sand mass in the case of mortars and in the proportion of 0–20% of the sand mass in the case of concretes [1]. The results of the tests conducted indicate that basalt powder can be used in the production of cement mortars and concretes as a substitute for natural sand [1]. In the area of autoclaved bricks, basalt components were added in the form of aggregate [16,17], basalt fiber [17,18,19,20] and basalt powder [21,22]. However, these studies focused primarily on autoclaved aerated concrete (AAC) bricks and not on silicates. Scientific cooperation with industrial production plants producing autoclaved bricks—silicates shows that these plants are looking for an additive or filler for bricks that will be ecological, will be characterized by a high content of silica in the composition and a low price (with the potential to reduce the price of ready-made bricks). Basalt dust is a component that meets the above requirements and has a large implementation potential.

In Europe, silicate products have been a valued and widely used building material for many years. More than 50% of European residential buildings are made of silicates. In Poland, for many years, silicates were an extremely underestimated and rarely used building material. Despite over 100 years of tradition and a dominant position in countries such as Germany, and especially the Netherlands, where this material “reigns” on the building materials market, sand-lime products have been rediscovered in our country only in recent years. Today, these products are becoming more and more popular among investors and designers.

The technical literature on the modification of sand-lime materials (silicates) states that the change in the composition and structure of the mixture is usually aimed at increasing the compressive strength of the finished product. The properties of sand-lime products depend on several important technological factors, mainly the quantity and quality of lime used in the silicate mix, the fineness of the mix components, the chemical composition of individual components, the amount of pressing pressure of the mix during the formation of semi-finished products, as well as the temperature, pressure and time of autoclaving of formed elements. By introducing additives (fillers) to the sand-lime mixture, new ions are often introduced, the quantity and quality of which have a significant impact on the formation of both the crystalline and amorphous C-S-H phase, as well as new phases not present in traditional products [23].

One of the effective ways of modifying sand-lime products in terms of improving their strength parameters is the use of ground mineral additives with a high specific surface area, pozzolanic and/or hydraulic properties, in the raw material mixture. An example of such a solution is the use of ground chalcedonite [24,25], ground limestone [26], dolomite powder [27], metakaolinite [28], barium [29], bentonite [30], thermally activated Carboniferous shale [31] and silica fume [32]. In recent years, the impact of waste glass in silicates has been extensively studied [33,34,35,36,37].

Known from the literature and patent descriptions are modifications of the composition of the sand-lime mixture, in which the mass substitute is mineral aggregates, i.e., barite [38,39,40], basalt [38], graphite [41] and igneous rocks of natural origin—preferably diabase [42].

Preliminary studies show an improvement in the physico-mechanical properties of sand-lime products with the addition of basalt powder [21]. This work is devoted to the study of the impact of waste material in the form of basalt powder on the parameters of silicate brick and the possibility of its management in the production of sand-lime products. The mixture of sand and lime was treated as one factor, because in the industrial conditions in which the tests were conducted, such a mixture was provided by the production plant. The mixture of sand and lime had constant proportions of both components. Basalt powder was a fine filler, the use of which in a very large amount would result in an increase in the water demand of the mixture, changes in the consistency of the mixture, problems during molding and, as indicated by preliminary tests [21], a general deterioration of the product properties.

## 2. Materials and Methods

### 2.1. Components of Raw Material Mass

As already mentioned in the introduction, the main components of autoclaved bricks (silicates) are sand, lime and water. In this work, in addition to the basic ingredients of the silicate raw material mass, basalt powder was used. This section describes the parameters and origin of the raw materials used in the research.

The lime used in the technological process comes from Trzuskawica S.A. Plant located in Sitkówka, Poland (the chemical composition of lime is given in Table 1) and it was a lime denoted as CL 90-Q, R5, P1, the main component of which is CaO.

The sand used for the tests came from Industrial Silicate Production Plant (Ludynia, Poland): H + H Silikaty Sp. z o. o. The plant extracts sand locally. The chemical composition of sand used in this study is given in Table 1.

According to PN-EN 1008:2004 [43], potable water, which is not subject to additional quality tests, is readily suitable for the production of silicate products. Such water was used.

The mineral powder used in the tests was a waste generated in the process of producing a mix of hard aggregate in the mixing plant, which was used for the production of MMA mineral-asphalt masses. This powder was obtained in the process of drying aggregates at a temperature of approx. 200 °C. It was captured in the fabric filter of the coating machine and then collected in a special tank. For the production of mineral-asphalt masses, hard mineral aggregate is used, which comes mainly from mines, which means that the resulting powder—treated as waste—has properties similar to the rocks from which it was formed. The mineral powder used in the research came from the crushing of basalt rocks.

Table 1 show chemical composition of basalt powder in comparison with sand and quicklime.

### 2.2. Production Technology

The production of sand-lime products can be divided into several stages, specifying the delivery and storage of raw materials, preparation of the lime-sand mixture, and the forming of products and autoclaving, which is the most important process in production. The mixing of sand and lime can be carried out before or during lime slaking. The lime-sand mixture, after the hydration process and possible enrichment with additives and/or admixtures, is fed into the molding of products in the press, usually at a pressure of 20 MPa. The task of autoclaving is to ensure optimal thermal and humidity conditions, in which a chemical reaction between the binder (lime) and the aggregate (sand) takes place. The product of this process is the formation of low-basic hydrated calcium silicates, which determine the final quality of the material. The prefabricated elements introduced into the autoclave are initially heated with saturated steam until the required hydrothermal conditions are reached. The actual autoclaving process is carried out at a temperature above 180 °C to 203 °C, using steam supplied from the boiler with a pressure of 0.8 to 1.6 MPa, respectively. The hydrothermal treatment time is longer the lower the working pressure is used, and ranges from 8 h for autoclaving at 1.6 MPa to about 12 h at 0.8 MPa steam pressure.

### 2.3. Planning of Experiment

There are many methods of planning experiences and sometimes it is difficult to choose the right one. This is due to the presence of many factors and variables affecting the production process and the selection of statistically significant ones for the analysis. In the case of silicates, the technological factors affecting the results of the research conducted include autoclaving time of individual stages, pressing conditions, quality of ingredients, autoclaving conditions and the share of individual ingredients in the raw material mass.

After analyzing the different experimental design methods available in the STATISTICA software (version 10.0, StatSoft Polska Sp. z o.o., Cracow, Poland) (bivalent fractional designs, trivalent fractional designs, central compositional designs, mixture designs, etc.), designs for limited mixture areas were selected. This is a special group of plans that allow you to analyze mixtures of compositions that add up to a constant value and take into account the quantitative limitations of the individual factors. A commonly used way of presenting the shares of mixtures, most often composed of no more than three components, are graphs in triangular coordinates. For example, consider a mixture of three components A, B, and C. Any mixture of three components can be uniquely specified by specifying a point in a triangular coordinate system defined by three variables.

**Figure 1:**
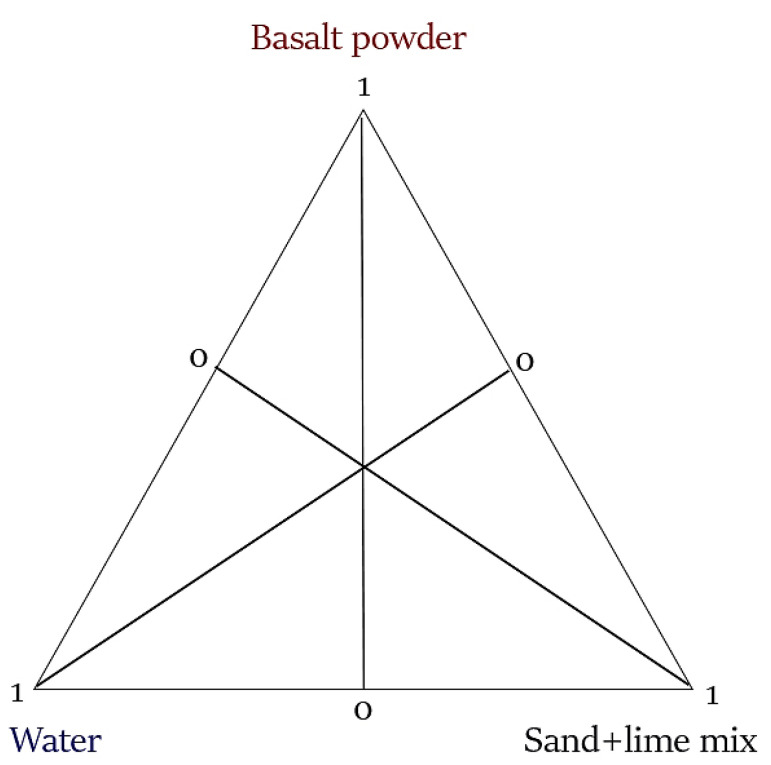
Model design of a mixture experiment with three independent variables.

The sum for each mixture is 1, so the values of individual components can be interpreted as contributions of the component (Figure 1). If the above data is represented as points on a three-dimensional graph, it turns out that these points formed a triangle in three-dimensional space. Valid mixtures are only the points inside the triangle where the sum of the component values is equal to 1. Therefore, you can limit yourself to drawing a triangle in order to clearly determine the values (shares) of individual components for each mixture.

The basic plans for mixtures assume that it is possible to design a mixture in which 100% of the share will be one of the ingredients and the others will have 0% shares. In the case of tests on sand-lime products that were prepared, this condition could not be met. Individual components have minimum and/or maximum share limits in the raw material mass, which enables appropriate reactions between the components during their mixing and autoclaving. For the selected plan for mixtures with restrictions, identical technological and material parameters were guaranteed during the tests, and only the composition of the silicate mixture was variable. As a result, 3 factors were obtained as input quantities—individual components of the mass: basalt dust (x_1_), water (x_2_), and a mixture of sand and lime (x_3_). Limitations for individual components are presented in Table 2.

Basalt powder was used in the proportion of 10–30% and water 5–15%. The use of a minimum 5% mass share of water in the composition of the mixture is necessary due to the chemical reactions that take place in the products at the production stage. On the other hand, the use of a proportion of water greater than 15%, although it is possible at the stage of mixing the raw masses, is unjustified, because during the pressing of the bricks, excess water flows out of the molds. Due to the limitations of basalt powder and water, the amounts of sand and lime was properly calculated to make the sum of the components equal to 100%.

**Figure 2:**
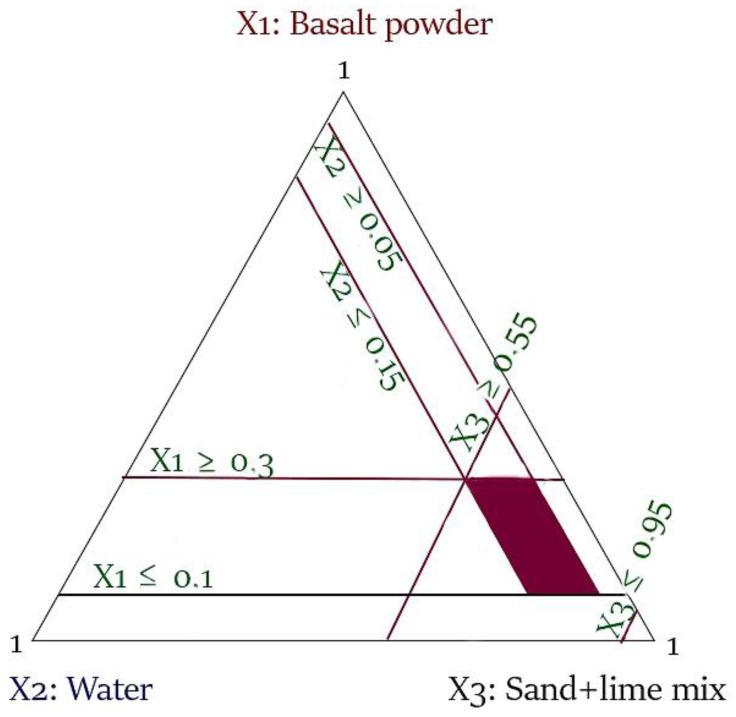
Model of a mixture experiment design with constraints for three independent variables.

The imposition of multiple constraints in a linear form for all independent variables (x_1_, x_2_, x_3_) in order to determine their appropriate combinations is shown in Figure 2.

**Figure 3:**
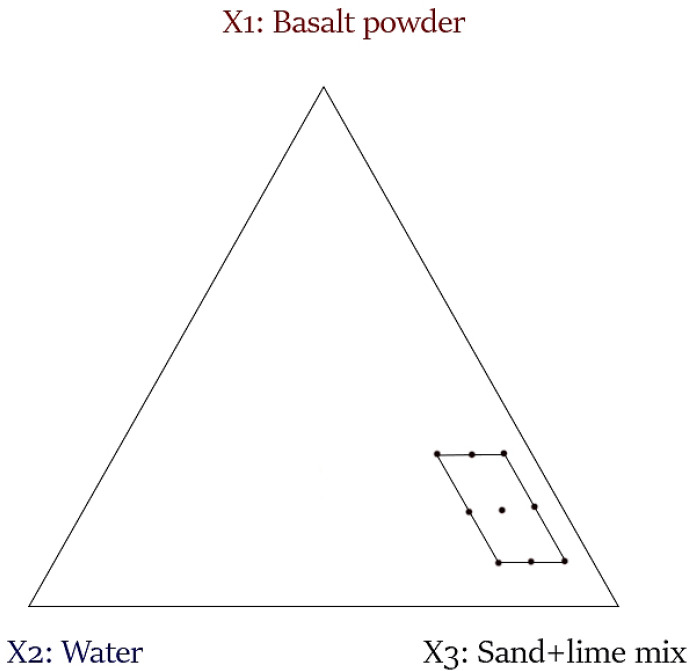
Experiment plan of mixtures with constraints—vertices and centroids generated for the tested range.

The dark area marked in Figure 2 determines the possible values of individual factors and their combinations under the imposition of the given restrictions of these factors. Piepel’s [44] and Snee’s [45] algorithms were used to determine the vertices and centers of gravity of the selected area. The results of the calculations are presented in Figure 3. Table 3 shows that 9 experimental systems were generated.

The “BP” marking symbolizes a percentage by weight of basalt powder in samples and “W”—the percentage of water in the raw material mass. For example, the name “BP10-W5” means a sample made of traditional raw material with the addition 10% of basalt powder and 5% of water.

### 2.4. Tests

The research was carried out in the laboratories of the Kielce University of Technology, in the H + H Silikaty Sp. z o.o. and in cooperation with the Polish Ceramic Society. The analysis of the research results was carried out using the STATISTICA software and Design Expert software (version 11, Stat-Ease, Inc., Minneapolis, MN, USA).

Experimental studies of individual compositions of sand-lime products were carried out on samples with dimensions of 40 × 40 × 160 mm. Before testing, the samples were stored for a minimum of 14 days in the temperature and air humidity conditions of ≥15 °C and ≤65%, respectively. Six samples of each type of product with the same composition were tested.

**Figure 4:**
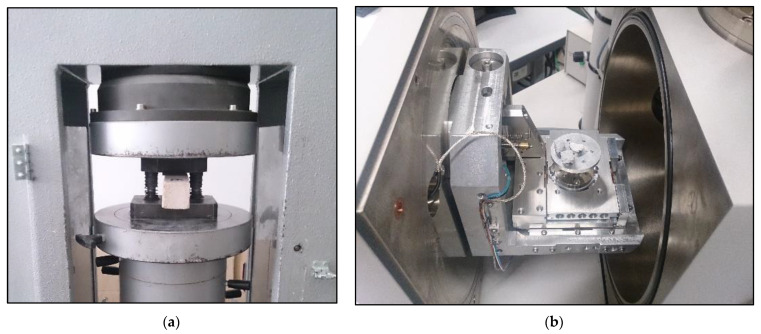
Equipment used during the tests: (**a**) compressive strength testing machine; (**b**) scanning microscope.

Compressive strength is the greatest stress that a sample of the test material can withstand when compressed. The numerical value of the compressive strength is the quotient of the compressive force that caused the destruction of the material structure and the surface on which the compressive force acts. Samples with a face surface of 40 × 160 mm were placed on the plate of the testing machine coaxially with the center of the pressure plate with dimensions of 62.5 × 40.0 mm, and then compressed uniformly until the sample was destroyed. The arithmetic mean of six measurement results on samples of identical composition was taken as the determination result. The compressive strength was determined on a compressive strength testing machine, in accordance with the guidelines of PN-EN 772-1: 11 + A1: 2015-10 [46]. Using a Tecnotest KC 300 compressive strength testing machine (Tecnotest, Treviolo, Italy, Figure 4a), the samples were compressed in a uniform manner until they were destroyed.

The density of the samples was tested on the basis of the PN-EN 772-13: 2001 standard [47]. Six samples of each product type were tested. Based on these measurements, the arithmetic mean of the density of each type of product was calculated. The density test was carried out using an ULTRAPYC 1200e helium pycnometer. The principle of operation of the apparatus is based on the use of gas to precisely determine the volume of the sample. The volume of the solid is that part of the previously calibrated measuring chamber that has not been occupied by gas. The mass of the sample is determined by weighing on an analytical balance and entered into the program operating the device.

The water absorption test was carried out in accordance with the guidelines set out in PN EN 772-11: 2011 [48] and PN-EN 772-21: 2011 [49]. Six samples of each type of product were dried to constant weight in a circulating air oven at 105 °C ± 5 °C, and then, after cooling, placed in a tank with water and left for 24 h. After this time, the samples were removed from the water and the tests were carried out.

Observations of the structure with optical microscopy were performed with the use of the Motic SMZ-168TP optical stereoscopic microscope (MOTIC, Xiamen, China). The maximum magnification of the microscope was 50×. Using the Moticam-3 camera compatible with Motic SMZ-168TP microscope digital photos of the samples were taken. Optical studies of the structure using an optical microscope were carried out for all samples. The material for the tests was obtained from the elements that remained after the destruction of the samples in the compressive strength tests. In order for the observations to be as accurate as possible, elements with the flattest surface were selected. The insides of the samples were analyzed, not the sides that were in contact with the press during brick formation.

The analysis of the microstructure of the obtained products was possible thanks to the use of the FEI Quanta FEG 250 scanning electron microscope (FEI, Brno, Czech Republic, Figure 4b), equipped with a detector measuring the energy of X-rays, EDS for short (Energy Dispersive Spectrometry, FEI, Hillsboro, OR, USA) or EDX (Energy Dispersive X-ray analysis, FEI, Hillsboro, OR, USA). Measurements were carried out in low vacuum conditions (water vapor pressure equaled 30 Pa) on non-sputtered samples. Magnifications from 150 to 20,000× were used. The tests were performed on fractures (shards) of the samples.

The phase composition of the obtained samples was determined using a PANalytical X-ray diffractometer, model Empyrean (Panalytical, Almelo, Netherlands). The share of individual phases was determined by the Rietveld method. Measurements were made using monochromatic radiation with a wavelength corresponding to the emission line K_α1_ of copper, in the angular range of 5–90° on a 2θ. The results were based on the ICDD database (The International Center for Diffraction Data). The analysis was carried out using the Deby—Scherrer—Hull (DSH) powder method on samples taken from samples whose compressive strength was closest to the average compressive strength value.

## 3. Results and Discussion

### 3.1. Compressive Strength

**Figure 5:**
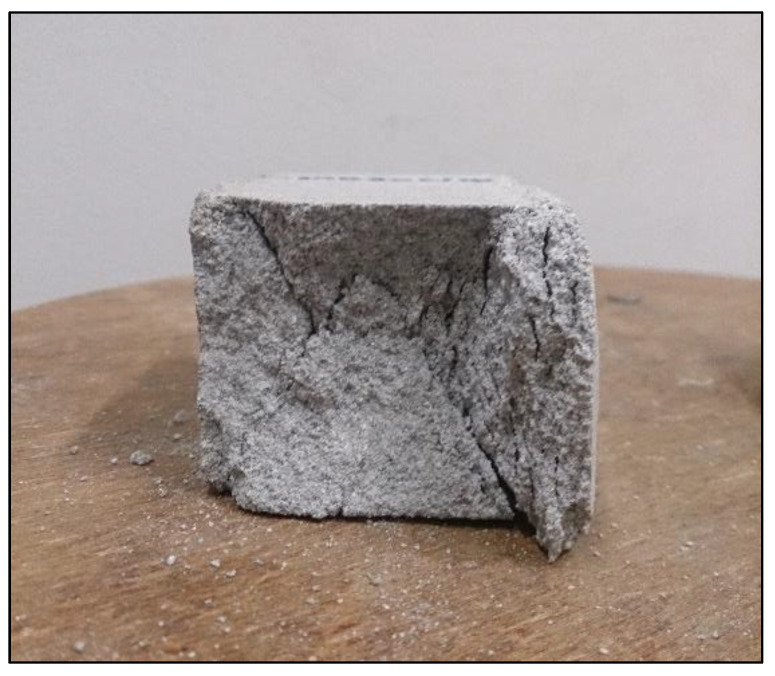
Cross-section of the sample as a result of compression.

Compressive strength is a particularly important property of sand-lime products, due to the fact that they are mainly used in the form of bricks for the construction of wall building elements. During strength tests, the samples were destroyed, but partially retained their structure. Visible during the tests was the characteristic hourglass shape appearing on the samples during the progress of loading (Figure 5). Although silicate is considered a brittle material, the samples after the end of the destructive tests still formed one element.

**Figure 6:**
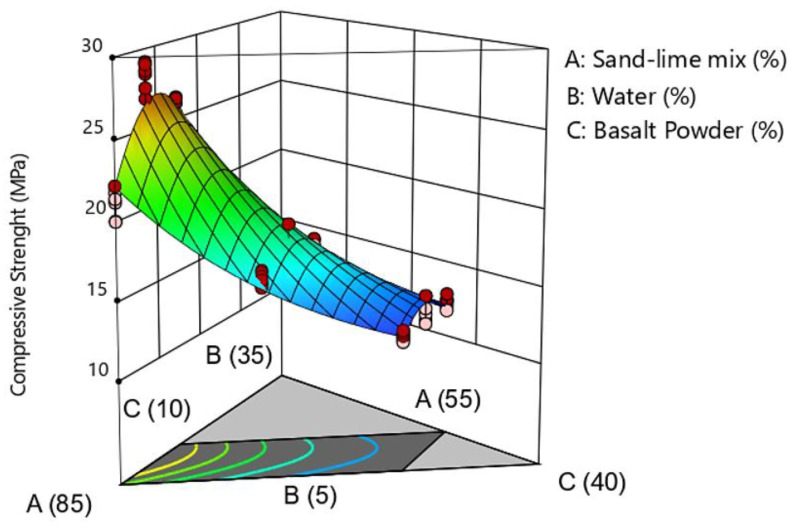
Compressive strength test sample.

**Figure 7:**
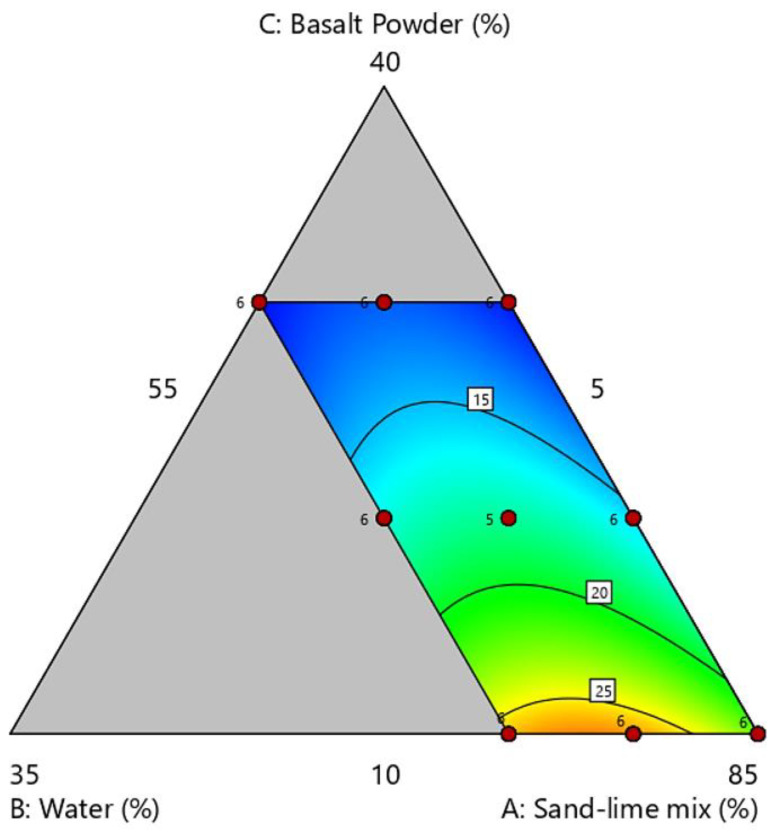
Ternary graph of compressive strength.

Spatial and contour plots (Figure 6 and Figure 7) were used to analyze the results of compressive strength tests. The models considered are presented in the form of graphs in a triangular coordinate system. Figure 6 shows the spatial distribution of compressive strength depending on the number of individual components in the raw material mixture.

On the triangular contour plot (Figure 7), the vertices and centers of gravity calculated in the experiment plan, which correspond to the compositions of the research recipes, were marked. With the increase in the share of basalt powder in the raw material mixture, the compressive strength decreased. The best results were obtained for the addition of basalt powder in the proportion of about 10% by weight. The BP10-W10 sample obtained a strength of slightly over 28 MPa in the tests. The amount of water in the mixture is also important. The graphs show that increasing the share of water in the silicate mass has a positive effect on the tested parameter.

Sand consists mainly of the sand fraction (about 95% are grains with a size of 100–1000 μm, and half of the basalt meal consists of powder and sand fractions. The addition of basalt meal increases the amount of powder and clay fractions in the raw material mass. Samples with basalt powder, thanks to the addition of fine grains, obtain a compact structure, which increases the density of the samples, and thus increases the compressive strength.

Although too much basalt powder causes a decrease in compressive strength. Nevertheless, all analyzed samples reached a strength higher than 11 MPa. The standards allow the use of bricks whose compressive strength is greater than 5 MPa, and standard silicates have strengths between 10 MPa and 15 MPa.

### 3.2. Water Absorption

**Figure 8:**
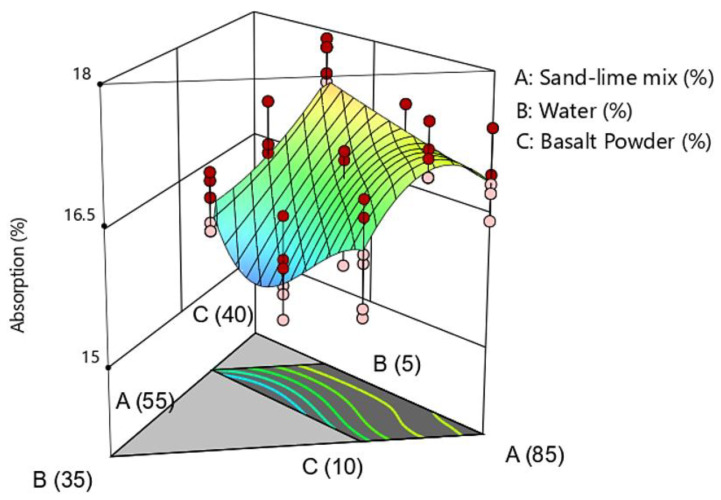
Water absorption of samples.

**Figure 9:**
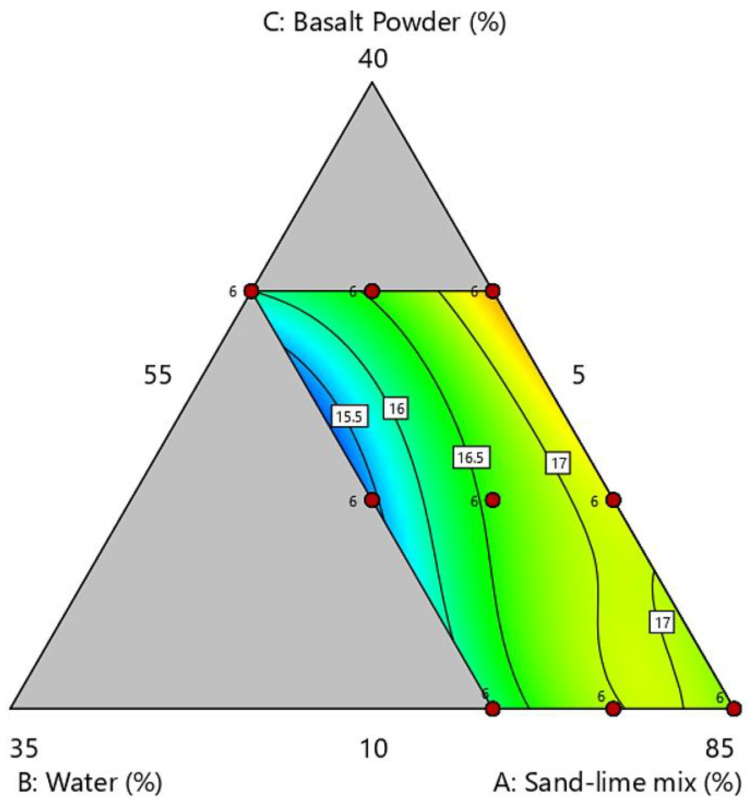
Ternary graph of water absorption.

The test results of water absorption are shown in Figure 8 and Figure 9. Standard sand-lime products achieve water adsorption of 16%. The results obtained in the tests are similar to standard silicates. Water adsorption of 17.5% was noted in the BP30-W5 sample.

### 3.3. Density

**Figure 10:**
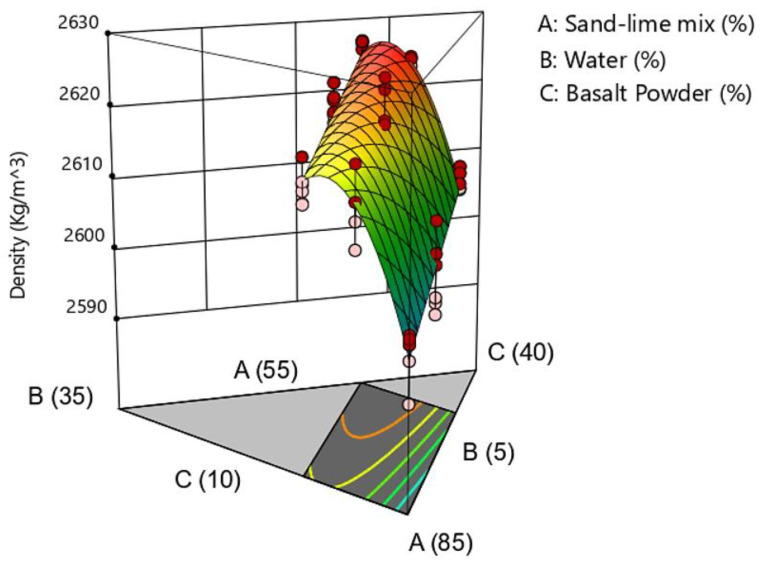
Density of samples.

**Figure 11:**
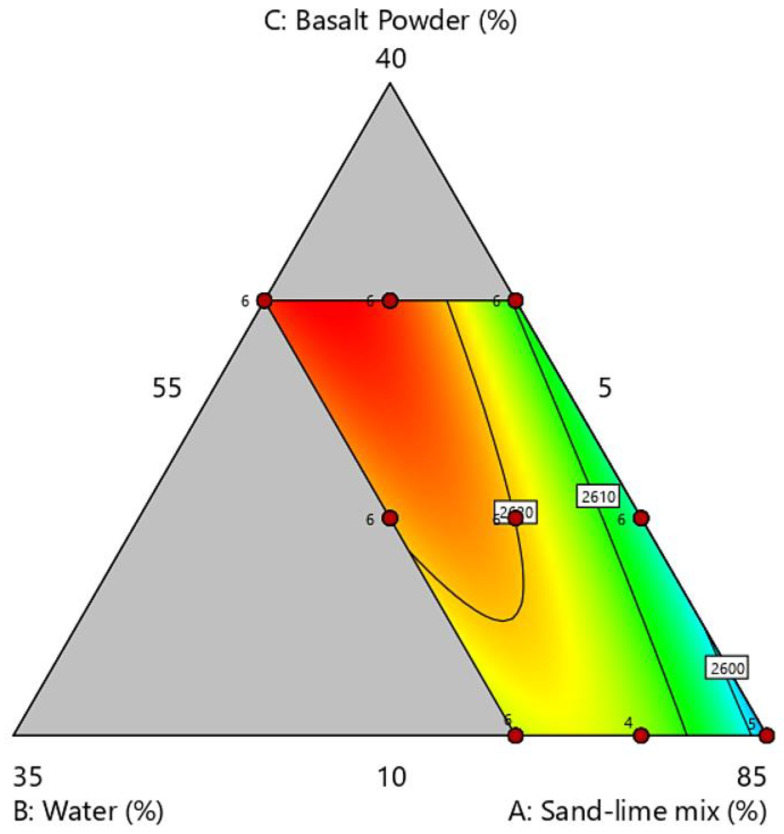
Ternary graph of density.

The results of the density tests are presented in the Figure 10 and Figure 11. The results of the density tests show a relationship between the share of basalt powder and the increase in density. As previously mentioned, basalt powder, due to its granulometry, fills free spaces in the mass and the products have a higher density. There was no significant difference in the products related to the water content of 10% and 15%. This may be due to the loss of some water during the sample compression stage. It was also a signal that a further increase in the water content above 15% in the samples is not possible with the production technology used in this case.

The increase in the density of the material in the case of silicates is a positive phenomenon, because it is usually associated with better thermal and acoustic insulation. Higher density is associated with an increase in the weight of the bricks. It cannot be unequivocally stated that this is a product defect, because it depends on the intended use of the product. Wall building materials with high density are used in residential construction due to increased acoustic insulation and in special construction, where high insulation of the wall is required, which will protect against, for example, X-rays diffraction (hospitals and laboratories).

### 3.4. Optical Microscopy

**Figure 12:**
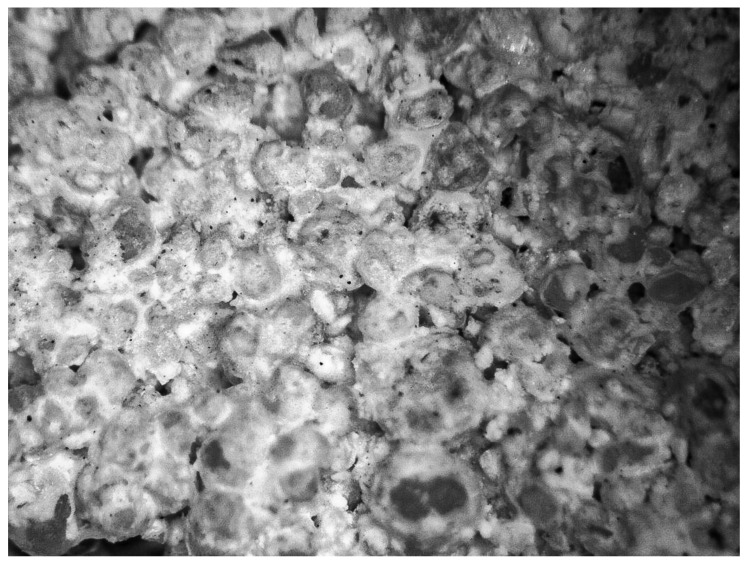
Optical microscope photo of the BP10-W10 sample.

Figure 12 shows a photo of the BP10-W10 sample structure. There were no significant differences between individual samples. Higher magnification is necessary to better analyze the structure of the samples.

In Figure 12, grains of sand of various sizes are visible. The grains are surrounded to varying degrees by lime and because of this they form an inseparable and hard element. Lime is visible in the photo in the form of light (white) structures. In Figure 12, there is a fragment of the sample, which shows various cases of enveloping of sand grains by lime. In the upper right part, the grains are covered to a lesser extent than in the rest of the tested sample. This is the result of insufficient homogenization of the mixture or an optical illusion caused by the camera and the darkening of this part of the photo.

### 3.5. SEM Scanning Microscopy

During autoclaving, basic chemical reactions occur, which determine the phase composition and microstructure of the autoclaved calcium silicate materials [50]. OH ions, after passing into the liquid phase, react with silicate ions, derived from the dissolution of SiO_2_ [23], resulting in the formation of hydrated calcium silicate, called C-S-H (calcium silicate hydrate) with different ratios of CaO, SiO_2_ and H_2_O. They are characterized by a different degree of structure order—from amorphous (the so-called “C-S-H phase”) to crystalline (tobermorite, xonotlite) [51,52].

**Figure 13:**
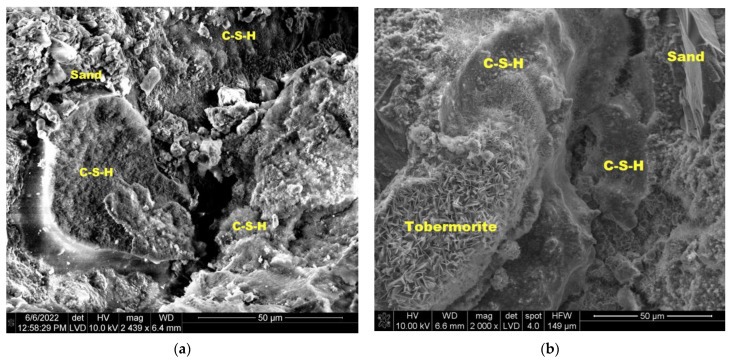
Microstructure of traditional sand-lime bricks; (**a**) industrial production; (**b**) laboratory production.

**Figure 14:**
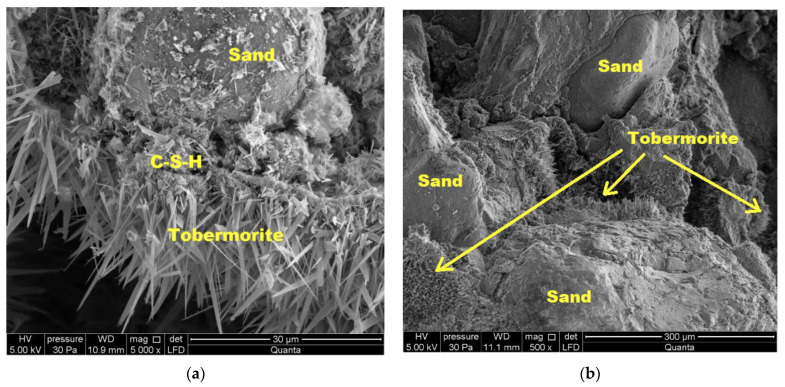
Microstructure of a BP10-W10 sample: (**a**) mag 5000×; (**b**) mag 500×.

**Figure 15:**
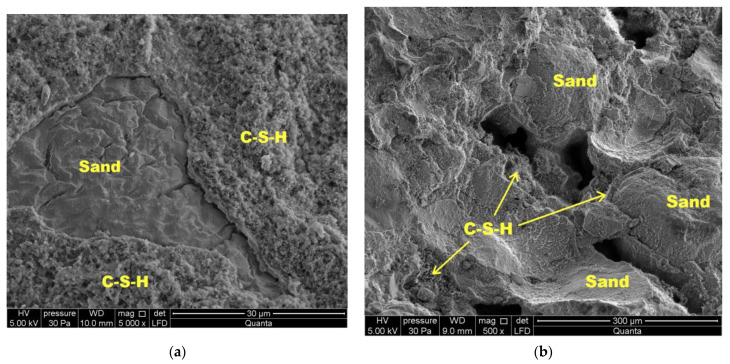
Microstructure of a BP30-W10 sample: (**a**) mag 5000×; (**b**) mag 500×.

There are many types of hydrated calcium silicates caused by the properties of the calcium atom, which with oxygen can form an octahedral coordination system with varying degrees of disorder. Therefore, these compounds are still the subject of numerous scientific studies. It is believed that hydrated calcium silicates with an ordered structure (crystalline phases) are formed from the amorphous C-S-H phase in hydrothermal conditions (at a temperature above 100 °C and a pressure above 0.1 MPa) [23,50,51,52]. The sequence of formation of individual phases is not accidental and is affected by the reactivity of the mixture components [51,52,53]. Hydrated calcium silicates are formed primarily on the surface of sand grains. Their nuclei are believed to form on the surface of the sand and then grow around the grains, filling the space occupied by water and dissolved anhydrous material as they grow. As a result, the free spaces between the grains are filled and the grains are bonded together to form a monolithic material with high strength [54]. It is estimated that with the increase in the number of synthesis products, especially the C-S-H phase and 1.1 nm tobermorite, the strength of the finished product increases [23,51,52], and the highest strengths are obtained when the reaction involves products with a molar ratio of C/S = 0.9–1.0 [52]. Figure 13, Figure 14 and Figure 15 show SEM images.

In traditional samples (from both industrial and laboratory production), the C-S-H phase and tobermorite were observed (Figure 13). In the BP10-W10 sample (Figure 14), xonotlite crystals are well developed and cover a large part of the sample. In the sample discussed, an increase in strength was observed compared to the other samples tested, which is also confirmed by the presence and structure of xonotlite.

Figure 15 presents SEM photos of the BP30-W10 sample, where much smaller amounts of xonotlite were observed. The dominant phase here is the C-S-H phase, which covers the sand grains. In the sample discussed, lower strengths were also noted compared to the BP10-W10 sample.

### 3.6. XRD Diffraction

Traditional silicates, due to the production process (high temperature and pressure above 1 Ba), consist mainly of crystalline phases. The dominant phase is the tobermorite phase. Depending on the type of modifier, however, crystallization may take different directions, which is caused by the chemical and mineralogical composition of the additive introduced to the raw material mass. In basalt powder, after the basic oxides such as SiO_2_, CaO, Al_2_O_3_, Fe_2_O_3_ and K_2_O, MgO, Na_2_O and SO_3_ are also present. Depending on the conditions and the amount of oxides, crystallization can proceed in the direction of natrolite, gyrolite, grucite, or the MSH phase. In the case of silicates, crystallization with such an amount of magnesium oxides proceed in the direction of brucite with a small share of the amorphous CSH phase. Depending on the course of crystallization of the CSH phase, it can transform into the MSH phase or the brucite phase, which is influenced by the content of magnesium oxide. The chemical composition of sand and basalt powder differs particularly in terms of the magnesium oxide content. The amount of magnesium oxide in the sand was below the measurable range. However, the chemical composition of the basalt powder (Table 1) shows that magnesium oxide constitutes even 10% of the composition of this raw material. The magnesium content in basalt can cause swelling of the aggregates, which is particularly noticeable in a humid environment (such conditions are also present in an autoclave). However, it depends on the amount of magnesium compounds in the material, the time of exposure to adverse weather conditions, or the quality of hydrothermal conditions (saturation with water vapor in an autoclave). In the tests presented and with the amount of the addition of basalt powder in the proportion of 10–30% by weight of the mass share, no process of swelling of the material was observed. Ph for such modified material is 10.7.

**Figure 16:**
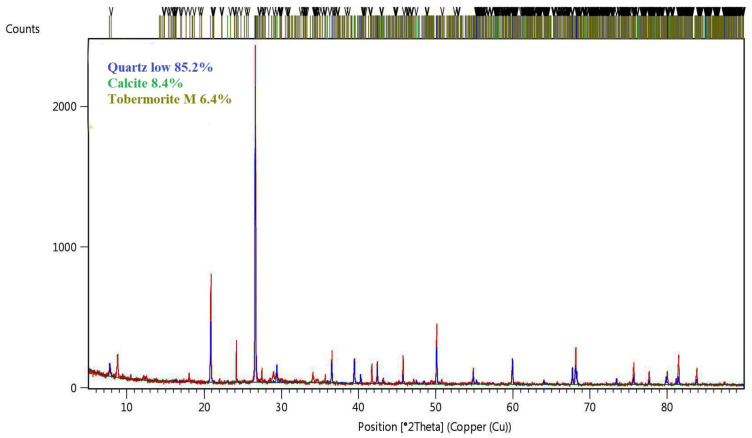
XRD analysis of sample BP10-W5.

Figure 16 shows the XRD analysis, while Table 4 standard molar thermodynamic properties of minerals in sand-lime bricks modified with basalt powder.

Admixtures and additives in the form of dusts and powders (in this case, basalt powder), due to their chemical properties and reactivity (related to the direction of crystallization), are of particular importance in the modification of building materials.

In order to determine these relationships, it is necessary to carry out a long-term analysis of the modified material and to determine the chemical reactions and phase transformations that occur over time. As a standard, in reference silicate materials, the dominant phase is tobermorite 11A.

The system of hydrated calcium silicates is also influenced by the concentration of carbon dioxide. According to the indicated studies, carbon dioxide gradually develops from adsorption to desorption on the surface of tobermorite 9 Å (001). These findings provide further insights into understanding the carbonation of hydrated calcium silicates.

### 3.7. Profiles for Predicted Values and Desirability

**Figure 17:**
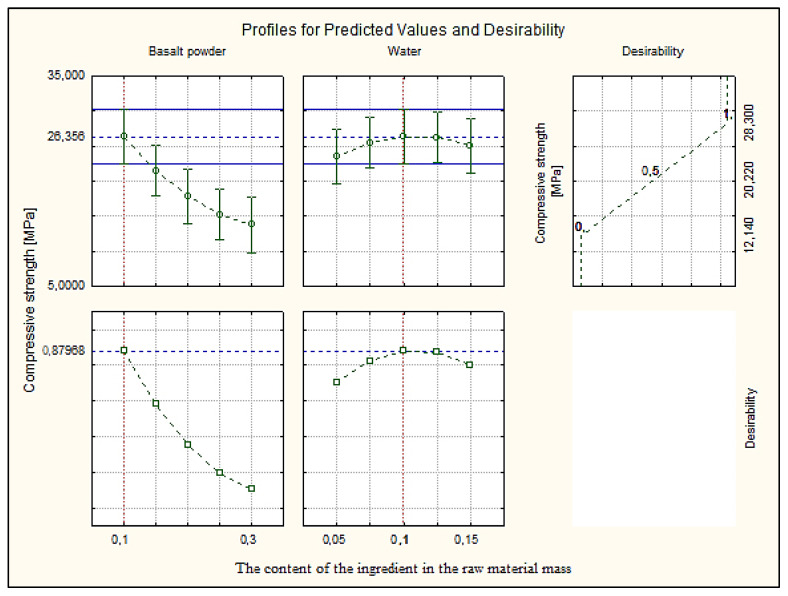
Profiles for predicted value and desirability for compressive strength.

**Figure 18:**
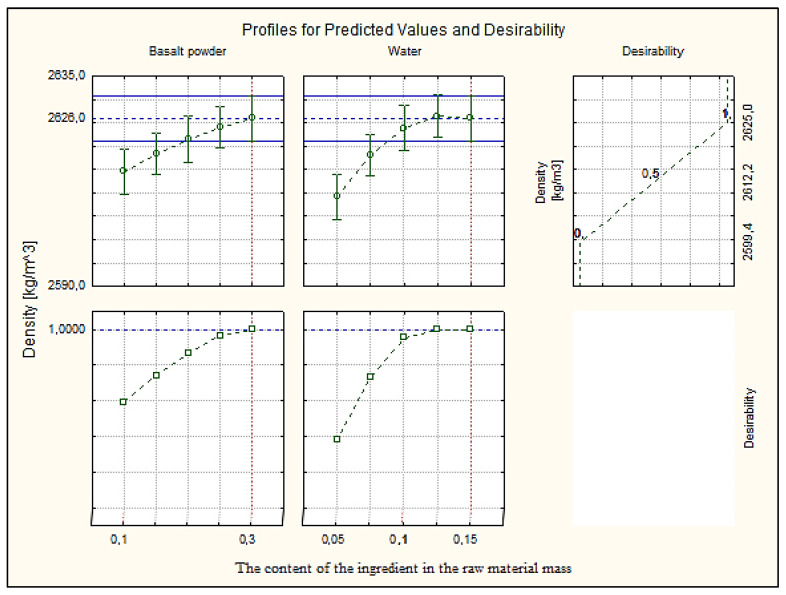
Profiles for predicted value and desirability for density.

**Figure 19:**
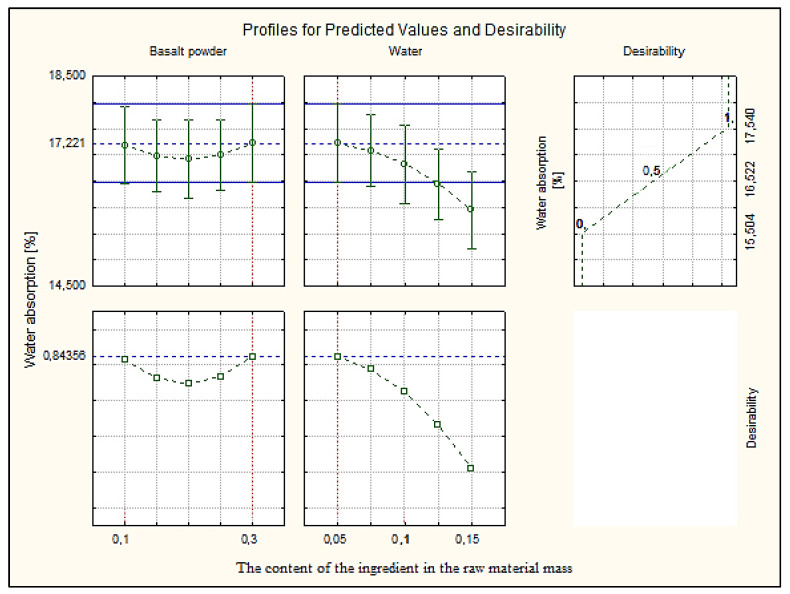
Profiles for predicted value and desirability for water absorption.

The paper presents the approximation and utility profiles for compressive strength (Figure 17), water absorption (Figure 18) and density (Figure 19). Once the appropriate actual input values have been selected, the approximation profile can be checked to see which input values result in the most desirable approximate output response.

The utility function determines the relative utility of different values of the output quantity. In order to determine the utility profile, we first determined the utility functions for each output quantity (dependent variable). For each of the optimized quantities, a response utility profile was created, where values from the range 0–1 were assigned in three points, where the digit 0 represents the least desirable (low) value, the digit 0.5 represents the average value, while the most favorable values of the examined variable were marked 1.0 (high).

The profiles of approximated values and their usefulness were created using the STATISTICA 10.0 computer program. The inputs were set at their expected optimal output values. The total utility was calculated as the geometric mean of the individual utilities for the predicted output values. Utility profiles consist of a series of plots (one for each output) of the total utility for various values of one output (independent variable), with the other inputs constant. By analyzing the response profiles, it is possible to determine which values of the input quantities lead to the desired responses of the output quantities.

The desired values of the output variables were determined, taking into account the design requirements of the modified silicate products. In Section 3.1, it was mentioned that silicate products with a compressive strength above 5.0 MPa, a bulk density above 2000 kg/m^3^ and an absorption of about 16% are sought. These values were the exponent to obtain such values of the output variables, in the utility profile for the approximated value.

Analyzing the profiles of approximated values and the utility in Figure 17, where the independent variables basalt powder and water were compared, the best results were obtained with the participation of 10% (0.1) by weight of basalt powder and 10% (0.1) by weight of water. The value of the dependent variable, compressive strength, is 26.36 MPa at the utility point of 0.88, i.e., in the range close to the most favorable value. Figure 18 shows the results for the dependent variable (bulk density) with the same values of the independent variables. The optimum value of about 2600 kg/m^3^ was obtained, i.e., significantly higher than the expected value at the utility point of 0.5. Such a value qualifies silicate products for use in order to increase the sound insulation of, e.g., walls. Figure 19 shows the dependent variable (absorption), with the same values of the independent variables as above. The absorption value is around 17%, i.e., the limit value, at the utility point of 0.63 (above the average value).

**Figure 20:**
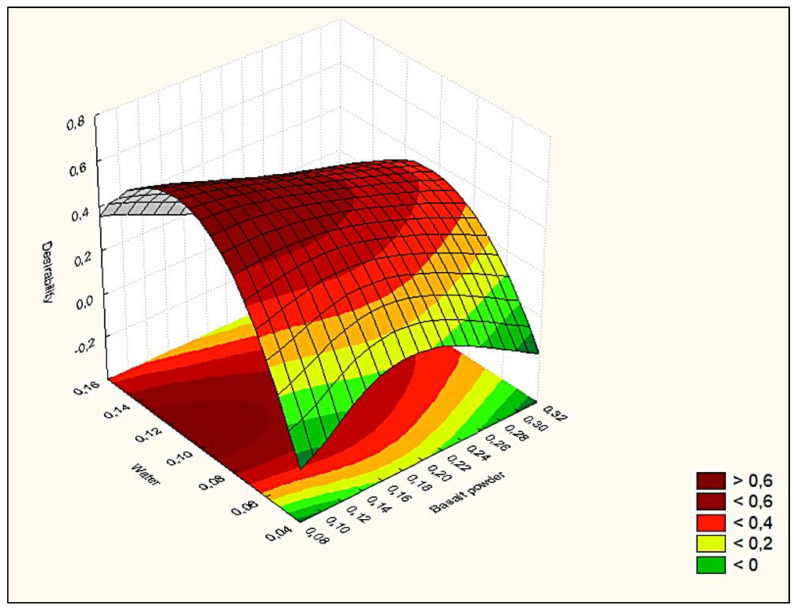
Desirability surface.

The research conducted in this work is aimed at the development of sand-lime products and the management of waste basalt powder. The product development process typically involves finding a set of conditions or input values that produce the most desirable product in terms of its properties or output response. Occasionally, quantities that maximize one dependent variable for another independent variable may have the opposite effect (Figure 20).

## 4. Conclusions

The paper analyzes the possibility of using waste in the form of basalt powder as a filler in autoclaved basalt materials, and its impact on the parameters of the modified product. Research clearly confirms that basalt powder can be used in the form of an additive to sand-lime products. In addition to ecology and protection of natural deposits, the advantage of this application is the economic aspect—reducing the cost of brick production resulting from the use of cheap post-production waste.

In order to eliminate the differences between the samples related to the technological conditions and the setting of the autoclave, the samples were made on the premises of the industrial silicate plant in Ludynia from one ready-made sand-lime mass, to which basalt powder and water were dosed. The best results were obtained for samples with a 10% mass share of basalt powder in the raw material mat and 10% water content (the remaining 80% by weight is a mix of sand and lime). For these samples, a compressive strength of 28 MPa was obtained, which is almost twice the typical values of silicates. Along with the increase in the use of basalt powder, a decrease in performance parameters was noted, but the products still met the conditions set for traditional silicate bricks. The deterioration of the properties of bricks with a higher content of basalt powder results from the decrease in the amount of binder—lime, the amount of which decreased in the mass inversely proportional to the amount of basalt powder. Powder was used as a filler, further research should be carried out to replace the appropriate fractions of sand with fractions of basalt powder and with different lime content. Such tests will allow the influence of basalt powder on sand-lime products to be fully determined.

The analysis of the microstructure of the samples indicates that the share of basalt powder affects the form of the tobermorite phase—according to the XRD analysis. In addition to the mechanical parameters of the autoclaving process (temperature, pressure), the quality and structure of the phases may be affected by the exploitation of the material and the concentration of CO_2_ in the atmosphere. On the BP10-W10 samples, tobermorite crystals formed—a phase considered to accompany the increase in strength in direct proportion to the increase in its amount in the sample tested. The results of compressive strength and material structure tests are correlated.

Further research will focus on the replacement of sand with basalt dust and the study of changes in samples related to this. The next stage of the research will also include an XRF (X-ray Powder Diffraction) analysis for thermodynamic simulation.

## Figures and Tables

**Table 1:** Chemical composition of basalt powder, sand and quicklime.

Oxides Composition	Basalt Powder [%]	Sand [%]	Quicklime [%]
SiO_2_	44.32	99.31	<QL
CaO	9.99	<QL	94.72
Al_2_O_3_	12.87	0.66	<QL
Fe_2_O_3_	11.51	0.27	<QL
MgO	10.01	<QL	0.97
Na_2_O	2.85	<QL	<QL
K_2_O	0.91	<QL	<QL
SO_3_	0.05	<QL	0.18
CO_2_	<QL	<QL	1.47
P_2_O_5_	0.45	<QL	<QL

QL—Quantifiable limit.

**Table 2:** Restrictions on the content of individual components in the raw material mass.

Component	Limitation
Basalt powder (x_1_)	x_1_ − 10 ≥ 0 −x_1_ + 30 ≥ 0
Water (x_2_)	x_2_ − 5 ≥ 0 −x_2_ + 15 ≥ 0
Sand-lime mix (x_3_)	x_3_ − 55 ≥ 0 −x_3_ + 95 ≥ 0

**Table 3:** Composition of individual samples.

No.	Sample	Ingredients		
		Sand-lime Mix	Water	Basalt Powder
		[%]	[%]	[%]
1	BP10-W5	85	5	10
2	BP10-W10	80	10	10
3	BP10-W15	75	15	10
4	BP20-W5	75	5	20
5	BP20-W10	70	10	20
6	BP20-W15	65	15	20
7	BP30-W5	65	5	30
8	BP30-W10	60	10	30
9	BP30-W15	55	15	30

**Table 4:** Standard molar thermodynamic properties of minerals in silicate bricks [55,56].

Mineral Name	Formula	Δ_f_G° [kJ/mol]	Δ_f_H° [kJ/mol]	S° [J/K·mol]	C°p [J/K·mol]	V^0^ [cm^3^/mol]	M [g/mol]
Quartz	Ca_0.8_SiO_2.8:1.5_4H_2_O	−1769.00	−1945.13	107.85	138.30	59.20	132.60
Tobermorite 11A	Ca_5_Si_6_H_11_O_22.5_	−9889.30	−10,680.00	692.50	764.90	286.10	739.90
Brucite	Mg(OH)_2_	−831.90	−924.10	59.43	77.27	24.63	58.32
Kaolinite	Al_2_Si_2_O_5_(OH)_4_	−3793.90	−4115.30	200.90	243.37	99.34	258.16

Δ_f_G°—standard molar Gibbs free energy of formation at T_o_ = 298 K; Δ_f_H°—standard molar enthalpy at T_o_ = 298 K; S°—standard molar entropy at T_o_= 298 K; C°p—heat capacity at T_o_ = 298 K; V°—molar volume; M—molar mass.

## Data Availability

The data presented in this study are available in Kostrzewa-Demczuk, P.; Stepien, A.; Dachowski, R.; Krugiełka, A. The use of basalt powder in autoclaved brick as a method of production waste management. *J. Clean. Prod.* **2021**, *320*, 128900, https://doi.org/10.1016/j.jclepro.2021.128900.

## Abbreviations

BP10-W5	sand-lime-basalt brick with 10% basalt powder and 5% water content
BP10-W10	sand-lime-basalt brick with 10% basalt powder and 10% water content
BP10-W15	sand-lime-basalt brick with 10% basalt powder and 15% water content
BP20-W5	sand-lime-basalt brick with 20% basalt powder and 5% water content
BP20-W10	sand-lime-basalt brick with 20% basalt powder and 10% water content
BP20-W15	sand-lime-basalt brick with 20% basalt powder and 15% water content
BP30-W5	sand-lime-basalt brick with 30% basalt powder and 5% water content
BP30-W10	sand-lime-basalt brick with 30% basalt powder and 10% water content
BP30-W15	sand-lime-basalt brick with 30% basalt powder and 15% water content
AAC	Autoclaved Aerated Concrete
SEM	Scanning Electron Microscope
XRD	X-ray Powder Diffraction [keV]
V	molar volume [cm3·mol^−1^]
M	molar mass [g·mol^−1^]
Log10K	solubility product at T = 298 K (25 °C)(this information is the basis for determining the pH of the material)
∆fG°	standard Gibbs free energy of formation [J·mol^−1^]
∆fH°	standard enthalpy of formation, is the change in enthalpy when one mole of a substance is formed from its elements under a standard pressure of 1 atm/1 bar [J·mol^−1^]
Cp°	specific heat [J·mol^−1^·K^−1^]
S°	standard entropy of formation [J·mol^−1^·K^−1^]
